# Dysregulated gene-associated biomarkers for Alzheimer’s disease and aging

**DOI:** 10.1515/tnsci-2021-0009

**Published:** 2021-02-05

**Authors:** Min Li, Rongxin Geng, Chen Li, Fantao Meng, Hongwei Zhao, Jing Liu, Juanjuan Dai, Xuezhen Wang

**Affiliations:** Department of Neurology, Binzhou Medical University Hospital, No. 661 Huanghe 2nd Road, Binzhou, Shandong, 256603, China; Department of Neurosurgery, Renmin Hospital of Wuhan University, Wuhan, Hubei, 430000, China; Institute for Metabolic & Neuropsychiatric Disorders, Binzhou Medical University Hospital, Binzhou, Shandong, 256603, China; Department of Neurosurgery, Binzhou Medical University Hospital, Binzhou, Shandong, 256603, China; Cancer Research Institute, Binzhou Medical University Hospital, Binzhou, Shandong, 256603, China

**Keywords:** Alzheimer’s disease, aging, differentially expressed genes, blood, hippocampus

## Abstract

Alzheimer’s disease (AD), the most common type of dementia, is a neurodegenerative disorder with a hidden onset, including difficult early detection and diagnosis. Nevertheless, the new crucial biomarkers for the diagnosis and pathogenesis of AD need to be explored further. Here, the common differentially expressed genes (DEGs) were identified through a comprehensive analysis of gene expression profiles from the Gene Expression Omnibus (GEO) database. Furthermore, Gene Ontology and Kyoto Encyclopedia of Genes and Genomes pathway analyses revealed that these DEGs were mainly associated with biological processes, cellular components, and molecular functions, which are involved in multiple cellular functions. Next, we found that 9 of the 24 genes showed the same regulatory changes in the blood of patients with AD compared to those in the GEO database, and 2 of the 24 genes showed a significant correlation with Montreal Cognitive Assessment scores. Finally, we determined that mice with AD and elderly mice had the same regulatory changes in the identified DEGs in both the blood and hippocampus. Our study identified several potential core biomarkers of AD and aging, which could contribute to the early detection, differential diagnosis, treatment, and pathological analysis of AD.

## 1 Introduction

Alzheimer’s disease (AD), the most common cause of dementia, has become an increasingly severe global public health concern, placing a colossal burden on both families and society [1]. AD is an age-related disease characterized by initial difficulties with memory, progressive cognitive impairment, dysfunctions in daily activities, and abnormal mental and behavioral changes. The cardinal pathological hallmarks of AD include intracellular neurofibrillary tangles and accumulation of extracellular amyloid-β conducive to senile plaques [2]. However, the lack of targeted biomarkers for diagnosis and pathogenesis was one of the key reasons for the current unavailability to treat or prevent AD [3].

Considerable evidence shows that the development of AD involves a genetic component and that the mutation or abnormal expression of genes triggers the occurrence and the progression of its core pathology [4]. Genetic analyses have determined four causal or genetic risk genes implicated in AD, such as amyloid-β protein precursor, presenilin 1 (PS1), PS2, and apolipoprotein E [5]. Clinically, the diagnosis of AD mainly relies on the history of individual cognitive and behavioral changes, opinions from other family members, cognitive tests, neurologic examinations, blood tests, and brain imaging ruling out other potential causes of dementia symptoms [6]. However, the novel pathological genes of AD require further exploration.

Age-related cognitive despair is a major risk factor for AD. Some genes or pathway deficits, such as insulin, IGF-1 signaling, and neuronal glucose transport, are involved in energy metabolism and inflammatory responses, and their connections are hallmarks of aging and neurodegenerative disorders such as AD [7]. In recent decades, microarray technology and gene expression profile analysis have become conventional means of exploring and screening differentially expressed genes (DEGs), thus enabling the identification of changes between patients and healthy individuals at the gene expression level. Molecular network analysis of the aging human frontal cortex has revealed co-expressed genes between aging and AD and its neuropathologic and cognitive endophenotypes [8]. Therefore, the identification of specific, sensitive, and reliable biomarkers and pathological genes is crucial for the early diagnosis and the therapeutic development of AD. Nevertheless, common functional genes in the blood and the brain involved in the pathology of AD and aging require further identification.

**Figure 1:**
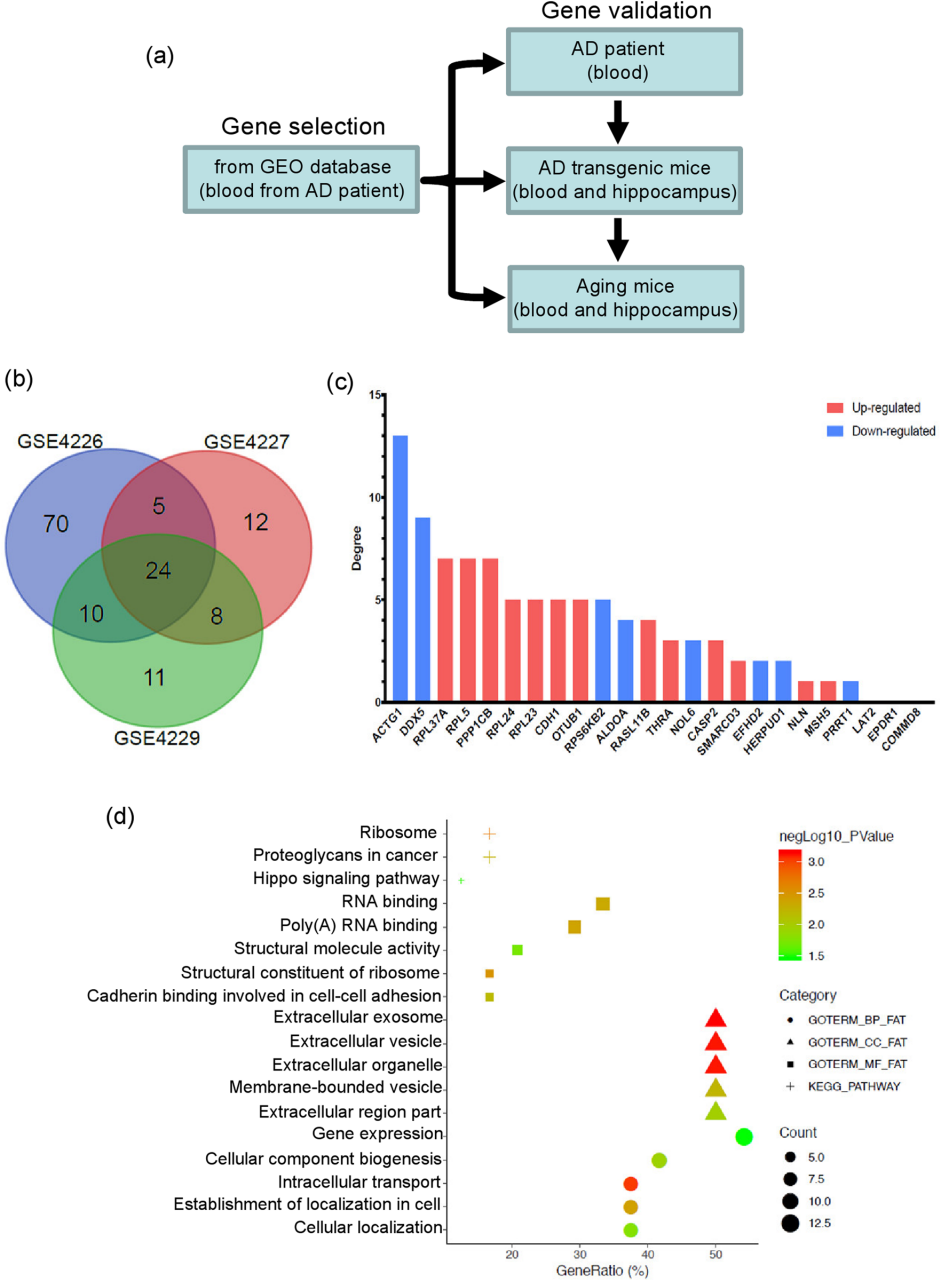
Analysis of differentially expressed genes in AD patients’ blood samples. (a) The framework of the whole study design. (b) The common DEGs derived from the Venn analysis of the three expression profiles (GSE4226, GSE4227, and GSE4229). (c) Expression levels of the common 24 DEGs in AD blood samples. The red column represents upregulated DEGs, and the blue column represents downregulated DEGs. (d) GO and KEGG pathway analyses of the DEGs.

In this study, we first analyzed the gene expression profiles of GSE4226, GSE4227, and GSE4229, which were selected from the Gene Expression Omnibus (GEO) database, to identify the key genes associated with AD. Then, GEO2R online tools and Venn analysis were utilized to analyze and identify the common DEGs. Furthermore, biological processes (BPs), cellular components (CCs), molecular functions (MFs), and the Kyoto Encyclopedia of Genes and Genomes (KEGG) pathways of the common DEGs were analyzed. Next, validation tests were conducted on human blood samples using quantitative real-time PCR (qPCR) to confirm the common DEGs, which may be served as the candidate biomarkers for AD early diagnosis. Finally, the expression of these DEGs was validated in the blood and hippocampus of AD and aged mice to identify the potential targeted genes for diagnosis and pathological researches of AD (Figure 1a).

## 2 Methods

### 2.1 Processing microarray data from the GEO database

Three databases (GSE4226, GSE4227, and GSE4229), which included the gene expression profiles of whole peripheral blood from patients with AD and normal elderly controls (NECs), were searched and obtained from the GEO database (http://www.ncbi.nlm.nih.gov/geo/) [9]. The numbers of subjects in each database were as follows: GSE4226: 14 AD and 14 NECs; GSE4227: 16 AD and 18 NECs; and GSE4229: 18 AD and 22 NECs. GEO2R, an open and shared interactive online tool, was utilized to identify DEGs between patients with AD and NECs (http://www.ncbi.nlm.nih.gov/geo/geo2r). The adjusted *p* < 0.05 and |log FC| > 1 were defined as the cutoff criteria. Venn analysis was utilized to identify the common DEGs. Gene Ontology (GO) and KEGG analyses were performed using the Database for Annotation, Visualization and Integrated Discovery (DAVID, http://david.ncifcrf.gov), an online bioinformatics database that consists of integrated biological data and stable analysis tools [10].

### 2.2 Subjects

Blood samples from patients with AD and NECs were collected from the Department of Neurology, Binzhou Medical University Hospital, Binzhou, China. The patients were diagnosed with AD based on the Montreal Cognitive Assessment (MoCA) scores and cortex and hippocampus atrophy detection results from magnetic resonance imaging (MRI); neurologic examinations were used to exclude other potential causes of dementia symptoms. Information regarding age, sex, disease duration, MoCA scores, and MRI results are summarized in Table 1. Blood samples were collected and stored immediately at −80°C.

**Table 1:** Information of 20 cases Alzheimer’s disease (AD) and 21 cases normal elderly controls (NECs)

Subjects	Gender	Age (YEARS)	Duration (YEARS)	MoCA	Hippocampus atrophy	Cortex atrophy
AD-1	Female	63	3	15	Yes	Yes
AD-2	Male	55	2	13	Yes	Yes
AD-3	Male	66	5	21	Yes	Yes
AD-4	Female	64	4	8	Yes	Yes
AD-5	Male	64	5	19	Yes	Yes
AD-6	Male	66	3	3	Yes	Yes
AD-7	Female	64	4	13	Yes	Yes
AD-8	Male	65	5	14	Yes	Yes
AD-9	Female	65	4	15	Yes	Yes
AD-10	Female	66	5	8	Yes	Yes
AD-11	Male	55	3	13	Yes	Yes
AD-12	Female	67	4	0	Yes	Yes
AD-13	Female	63	5	7	Yes	Yes
AD-14	Female	65	3	10	Yes	Yes
AD-15	Male	58	4	14	Yes	Yes
AD-16	Male	55	2	17	Yes	Yes
AD-17	Female	68	3	9	Yes	Yes
AD-18	Male	64	4	10	Yes	Yes
AD-19	Female	63	5	6	Yes	Yes
AD-20	Male	61	4	15	Yes	Yes
NEC-1	Male	68	0	27	No	No
NEC-2	Male	56	0	29	No	No
NEC-3	Female	64	0	28	No	No
NEC-4	Male	65	0	30	No	No
NEC-5	Male	62	0	28	No	No
NEC-6	Female	62	0	27	No	No
NEC-7	Female	58	0	30	No	No
NEC-8	Male	57	0	28	No	No
NEC-9	Female	65	0	27	No	No
NEC-10	Male	61	0	26	No	No
NEC-11	Female	68	0	29	No	No
NEC-12	Female	61	0	27	No	No
NEC-13	Female	55	0	27	No	No
NEC-14	Male	68	0	28	No	No
NEC-15	Male	66	0	26	No	No
NEC-16	Female	59	0	29	No	No
NEC-17	Female	62	0	26	No	No
NEC-18	Male	65	0	30	No	No
NEC-19	Female	68	0	28	No	No
NEC-20	Female	67	0	30	No	No
NEC-21	Male	66	0	28	No	No

### 2.3 Animals

Male wild-type (WT) C57BL/6J mice (Stock No. 000664) were purchased from the Jackson Laboratory (Bar Harbor, ME, USA) and maintained as a colony. APP/PS1 (Stock No. 004462) mice [11,12], which express a chimeric mouse/human amyloid precursor protein (APP) and mutant human presenilin 1 (PS1) in neurons, were obtained from the Jackson Laboratory. For genotyping, the following PCR primers were used: PS1: forward-5′-AATAGAGAACGGCAGGAGCA-3′, reverse-5′-GCCATGAGGGCACTAATCAT-3′; APP: forward-5′-AGGACTGACCACTCGACCAG-3′, reverse-5′-CGGGGGTCTAGTTCTGCAT-3′. All mice were housed in groups of five per cage under a 12 h light/dark cycle (lights on at 7:00 am) with *ad libitum* access to water and standard food pellets.


**Informed consent:** Informed consent has been obtained from all individuals included in this study.
**Ethical approvals:** (1) The research related to human use has been complied with all the relevant national regulations, institutional policies and in accordance the tenets of the Helsinki Declaration, and has been approved by the authors’ institutional review board (approval number: 2019-LW-009). (2) The research related to animals’ use has been complied with all the relevant national regulations and institutional policies for the care and use of animals.

### 2.4 Quantitative real-time PCR analysis

Mice were decapitated, and the blood and hippocampi were rapidly collected. Total RNA was extracted from the blood or hippocampi using TRIzol reagent (Invitrogen, Carlsbad, CA, USA) and purified according to the manufacturer’s recommendations. cDNA was synthesized using a RevertAid First Strand cDNA Synthesis Kit (K1622, Thermo Fisher Scientific, Waltham, MA, USA). The resulting cDNA was used for qPCR quantification using a StepOnePlus Real-Time PCR system (Applied Biosystems, Waltham, MA, USA), in accordance with the manufacturer’s instructions. Gene expression levels were normalized to those of GAPDH. The 2^−∆∆CT^ (cycle threshold) method was used to calculate and analyze relative mRNA expression levels [13,14,15,16,17]. All primer sequences are listed in supplementary Table S1.

### 2.5 Morris water maze

The Morris water maze (MWM) test was performed as described previously [18]. Briefly, the water maze (1.50 m in diameter and 0.50 m in height) was filled with water (20 ± 1°C; dyed white) to maintain the water surface height at 1.50 cm. The tank was divided into four quadrants. The hidden platform remained at a constant position throughout the trials and was placed at the center of either quadrant. Video tracking software was used to track the animals. Learning and memory acquisition lasted for 5 days. Animals were placed in the water at four points randomly every day until they reached the platform and remained there for 10 s within 1 min; otherwise, the mouse was manually guided to the platform. On the day, the learning and memory test was carried out, the platform was removed, and the mice were placed in the water in the opposite quadrant to where the platform was previously located, and the number of times the mouse crossed the platform’s previous location, total distance, and total time were recorded within 60 s.

### 2.6 Y maze

The Y maze (YM) test was conducted as described previously [19]. In brief, the apparatus for the YM test consisted of gray plastic (with each arm 40 cm long, 12 cm high, 3 cm wide, and 10 cm wide at the top) at the bottom. The three arms were connected at an angle of 120°. The mice were placed at the end of an arm and allowed to explore the maze freely for 10 min. The total arm entries and spontaneous alternation percentages (SA%) were measured. SA% was defined as the ratio of the arm choices that differed from the previous two choices (“successful choices”) to total choices during the test.

### 2.7 Statistical analysis

All statistical analyses were performed using the statistical software GraphPad Prism 7. Shapiro-Wilk test and *F* test were used to test the normality and equal variance assumptions, respectively. To compare two groups, two-tailed *t* tests were used for normally distributed data. Two-tailed *t*-tests with Welch’s correction were used with normally distributed data for unequal variances. Mann-Whitney *U* tests were used for the nonnormally distributed data. For escape latency in the MWM test, two-way repeated-measures ANOVA followed by Tukey’s test was used. The percentage of male and females in both normal and AD groups was analyzed by the Chi-square test. The linear relationships between two variables were determined by calculating Pearson’s correlation coefficient. *P* < 0.05 was considered as statistically significant. All data are presented as the mean ± standard error (s.e.m.).

## 3 Results

### 3.1 Identification of DEGs from the GEO database

We aimed to identify the DEGs by comparing the gene expression profiles of patients with AD and NECs. In the GEO database, three gene expression profiles (GSE4226, GSE4227, and GSE4229) were acquired by searching for AD of *Homo sapiens*, which was not really included in the previous studies. The DEGs were selected and detected using the GEO2R online tool with default parameters. Compared with the NECs, 61 upregulated and 53 downregulated DEGs were detected from GSE4226, 28 upregulated and 22 downregulated from GSE4227, and 38 upregulated and 15 downregulated from GSE4229. Venn analysis was utilized to handle three expression profiles (GSE4226, GSE4227, and GSE4229) and acquire 24 common DEGs, including 15 upregulated and 9 downregulated genes (Figure 1b and c). In addition, GO function and KEGG pathway analyses of the 24 common DEGs were performed using DAVID tools. The results indicated that the common DEGs were mainly enriched in the BP, MF, CC, and KEGG pathways. Common DEGs for BP accounted for gene expression, CC biogenesis, intracellular transport, establishment of localization in cells, and cellular localization. Common DEGs for the CC analysis were chiefly implicated in extracellular exosomes, extracellular vesicles, extracellular organelles, membrane-bound vesicles, and extracellular regions. Common DEGs for MF were mainly related to RNA binding, poly(A) RNA binding, structural molecule activity, structural constituents of ribosomes, and cadherin binding involved in cell–cell adhesion. KEGG pathway analysis demonstrated that common DEGs were enriched in the ribosomes, proteoglycans in cancer, and hippocampus signaling pathway (Figure 1d).

### 3.2 Verification of the identified DEGs in blood samples from patients with AD

**Figure 2:**
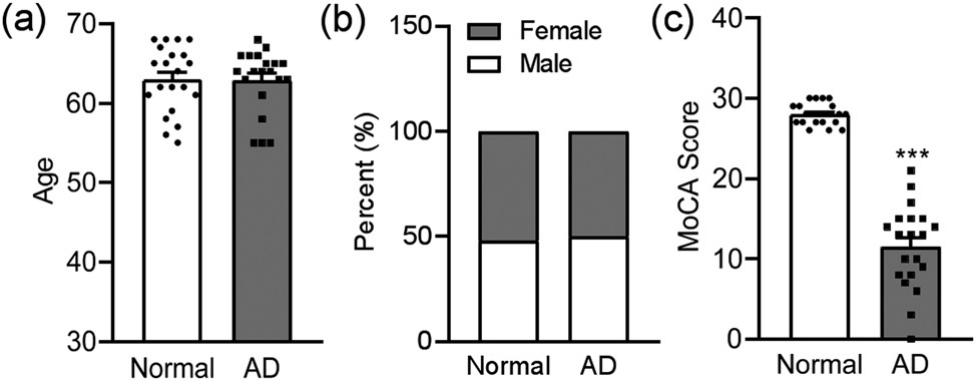
The age, sex, and MoCA scores of the patients. (a) Age, (b) sex, and (c) MoCA scores of patients with NECs and AD. NECs: *n* = 21; AD: *n* = 20. ***p < 0.001 compared to control subjects.

**Figure 3:**
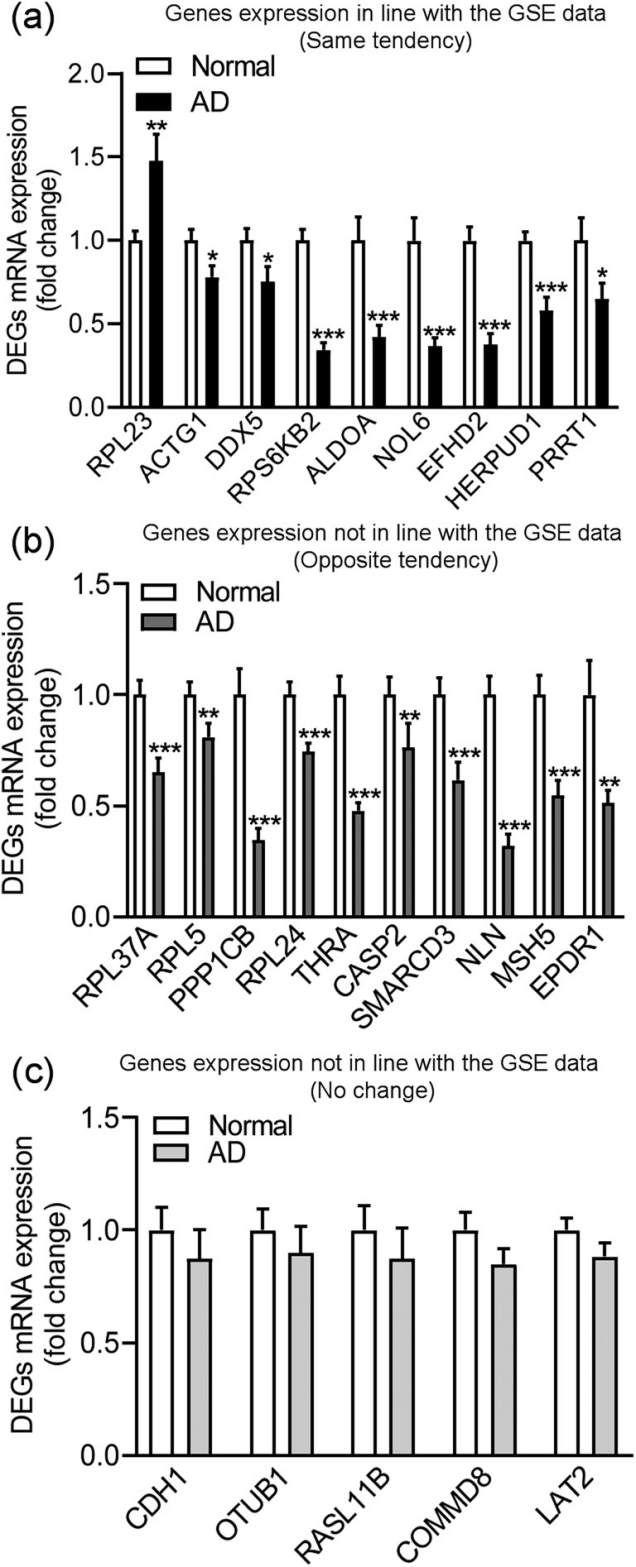
Detection of DEGs in the blood samples of patients with AD. (a) Significant changes in gene expression in line with the GSE data, (b) significant changes in gene expression in direct contrast to those in the GSE data analysis, (c) no changes in gene expression. NECs: *n* = 21; AD: *n* = 20. *p < 0.05; **p < 0.01; ***p < 0.001 compared to normal.

The identified DEGs were confirmed using the blood samples of patients with AD collected from our hospital. There was no statistical difference between those with AD and NECs in terms of age and sex (age: *p* = 0.8206; sex: *p* = 0.7773, Figure 2a and b), while these patients had significantly lower MoCA scores and evident cortex and hippocampus atrophy than the control subjects (*p* < 0.0010; Figure 2c and Table 1). Our qPCR results indicated that the mRNA levels of RPL23 (*p* = 0.0050) in blood significantly increased in patients with AD, whereas mRNA levels of ACTG1 (*p* = 0.0136), DDX5 (*p* = 0.0348), RPS6KB2 (*p* < 0.0010), ALDOA (*p* < 0.0010), NOL6 (*p* < 0.0010), EFHD2 (*p* < 0.0010), HERPUD1 (*p* < 0.0010), and PRRT1 (*p* = 0.0424) significantly decreased compared to those in the NEC group (Figure 3a), and the change in the expression of these genes was in line with the results of the GSE data analysis. Furthermore, the mRNA levels of RPL37A (*p* < 0.0010), RPL5 (*p* = 0.0084), PPP1CB (*p* < 0.0010), RPL24 (*p* < 0.0010), THRA (*p* < 0.0010), CASP2 (*p* = 0.0077), SMARCD3 (*p* < 0.0010), NLN (*p* < 0.0010), MSH5 (*p* < 0.0010), and EPDR1 (*p* = 0.0099) genes were significantly lower in the patients with AD than in the NEC group (Figure 3b), and the change in expression of these genes was in direct contrast to the results of the GSE data analysis. Nevertheless, there were no differences in the mRNA levels of CDH1 (*p* = 0.3189), OTUB1 (*p* = 0.4458), RASL11B (*p* = 0.4007), COMMD8 (*p* = 0.1035), and LAT2 (*p* = 0.1093) genes between patients with AD and the NECs (Figure 3c).

**Figure 4:**
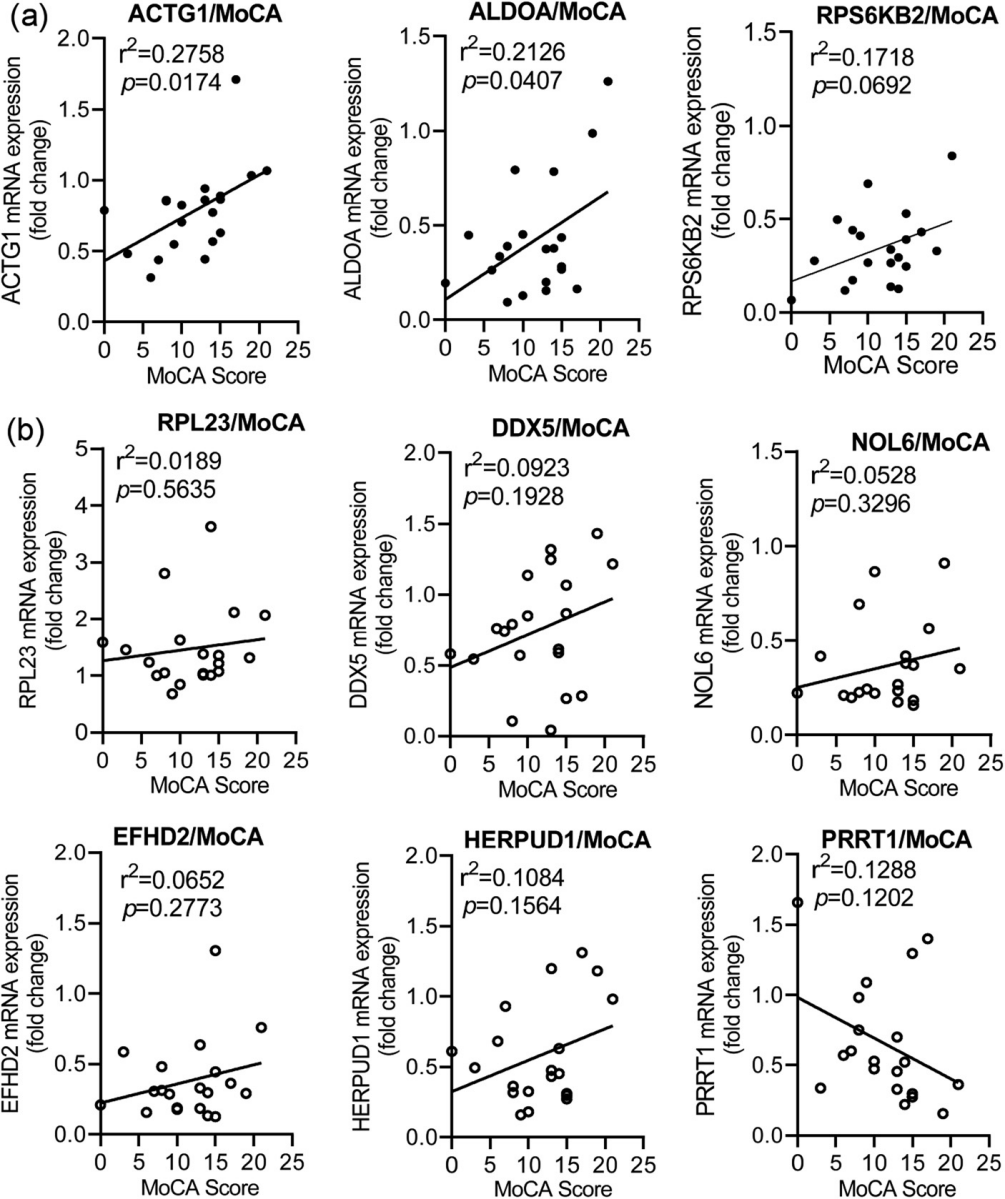
Correlation analysis of the nine identified DEGs with MoCA scores. (a) The genes showing significant positive correlation with MoCA scores. (b) The genes showing no significant correlation with the MoCA scores.

Correlation analysis was conducted between the mRNA expression levels of nine identified genes whose change in expression was similar to that observed in GSE data analysis and MoCA scores. We found that the expression levels of ACTG1 and ALDOA were positively correlated with MoCA scores, with the *RPS6KB2* gene showing a trend for significant positive correlation with these scores (Figure 4a). The other genes showed no obvious correlation with the MoCA scores (Figure 4b).

### 3.3 The expression profiles of the DEGs in blood samples from AD and aging mice

**Figure 5:**
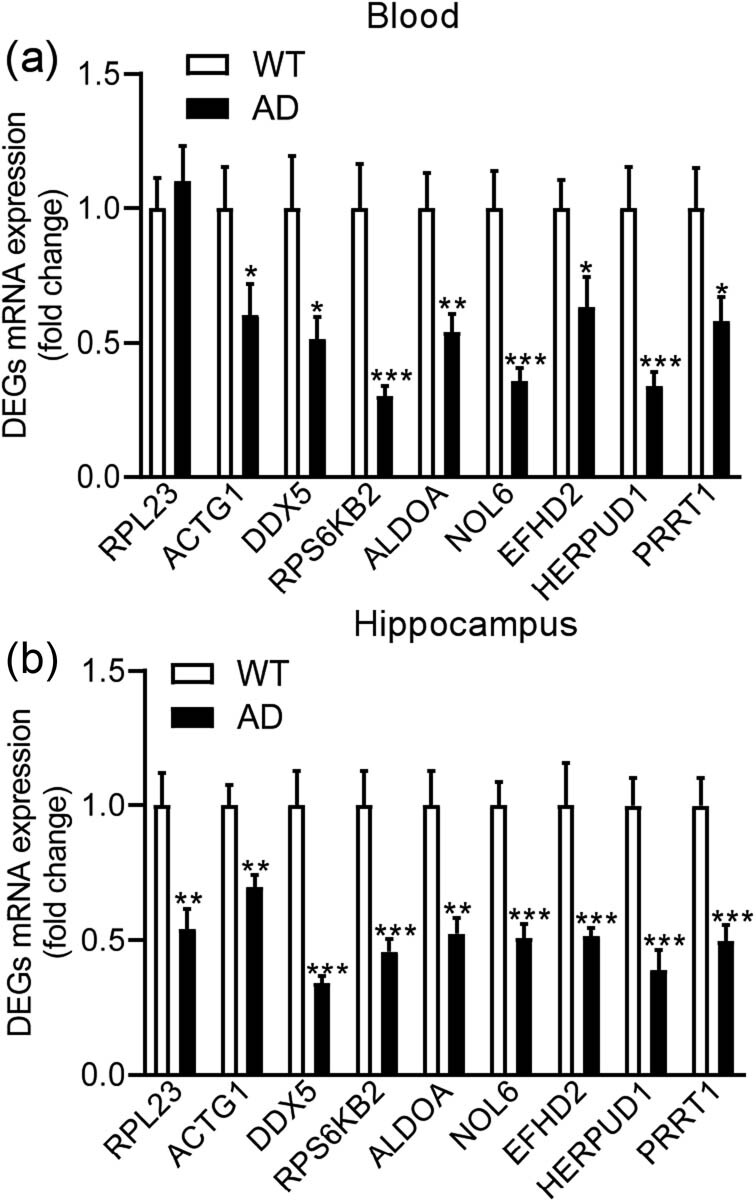
The mRNA expression levels of the nine identified DEGs in AD mice. The mRNA expression levels of the nine identified DEGs in the blood (a) and hippocampi (b). WT mice, *n* = 11; AD mice, *n* = 12; *p < 0.05; **p < 0.01; ***p < 0.001 compared to WT.

To further confirm the expression of the DEGs, we used AD transgenic mice (18 months old) with learning memory deficiencies [11] to test the expression profiles of the nine identified DEGs with a similar change in expression as that found in the GSE data analysis. The mRNA levels of all nine DEGs, except for RPL23, decreased in the blood of AD mice compared to those in the blood of WT mice (RPL23: *p* = 0.5657; ACTG1: *p* = 0.0188; DDX5: *p* = 0.0268; RPS6KB2: *p* < 0.0010; ALDOA: *p* = 0.0046; NOL6: *p* < 0.0010; EFHD2: *p* = 0.0188; HERPUD1: *p* < 0.0010; PRRT1: *p* = 0.0222; Figure 5a). Meanwhile, the expression of the nine DEGs in the hippocampi of AD and WT mice were also measured, and the results showed that the expression levels of all DEGs decreased in the hippocampi of AD mice compared to those in their control littermates (RPL23: *p* = 0.0034; ACTG1: *p* = 0.0021; DDX5: *p* < 0.0010; RPS6KB2: *p* < 0.0010; ALDOA: *p* = 0.0086; NOL6: *p* < 0.0010; EFHD2: *p* < 0.0010; HERPUD1: *p* < 0.0010; PRRT1: *p* < 0.0010; Figure 5b).

**Figure 6:**
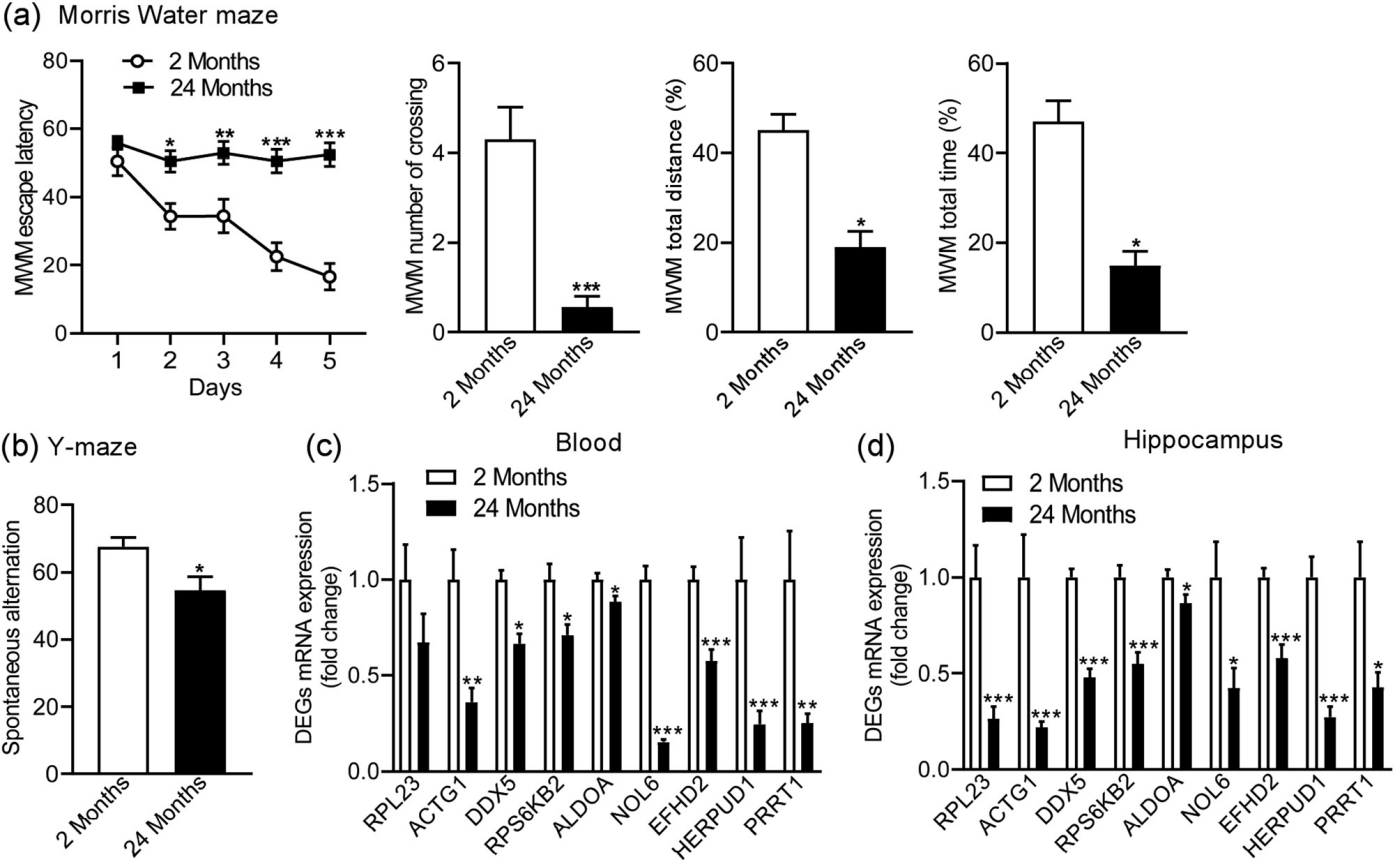
The mRNA expression levels of the nine identified DEGs in aging mice. Morris Water Maze (a) and Y-Maze tests (b) of 2- and 24-month-old mice. The mRNA expression levels of the nine identified DEGs in the blood (c) and hippocampi (d). 2 months, *n* = 10; 24 months, *n* = 9. *p < 0.05, **p < 0.01, ***p < 0.001 compared to 2 months.

Aging is an important contributor to the pathology of AD [20,7]. Therefore, we next analyzed the expression of these identified DEGs in the blood and hippocampi of aging mice. First, we evaluated the learning memory behaviors of the mice of different ages (2 vs 24 months), and the 24-month-old mice showed significantly higher escape latencies than the 2-month-old mice (age: F (1, 85) = 76.6400, *p* < 0.0010; days: F (4, 85) = 7.5480, *p* < 0.0010; age × days: F (4, 85) = 4.8400, *p* = 0.0015) in the MWM test. The crossing number, total distance, and total time taken by the 2-month-old mice decreased compared to that for the 24-month-old mice (*p* < 0.0010; *p* < 0.0010; *p* < 0.0010) in the MWM test (Figure 6a). Our results also revealed that the SA in the YM test of 2-month-old mice decreased compared to that of the 24-month-old mice (*p* = 0.0131; Figure 6b).

Next, we evaluated the expression of the nine DEGs with the similar change in expression as that found in the GSE data analysis. The results revealed that the expression levels of all DEGs, except for RPL23, decreased in the blood of the 24-month-old mice compared to those in the 2-month-old mice (RPL23: *p* = 0.1882; ACTG1: *p* = 0.0025; DDX5: *p* < 0.0010; RPS6KB2: *p* = 0.0112; ALDOA: *p* = 0.0249; NOL6: *p* < 0.0010; EFHD2: *p* < 0.0010; HERPUD1: *p* = 0.0059; PRRT1: *p* = 0.0140; Figure 6c). Meanwhile, the expression of the nine DEGs in the hippocampi decreased in the blood of 24-month-old mice compared to that in 2-month-old mice (RPL23: *p* < 0.0010; ACTG1: *p* < 0.0010; DDX5: *p* < 0.0010; RPS6KB2: *p* < 0.0010; ALDOA: *p* = 0.0472; NOL6: *p* = 0.0172; EFHD2: *p* < 0.0010; HERPUD1: *p* < 0.0010; PRRT1: *p* = 0.0140; Figure 6d).

## 4 Discussion

In the present study, 24 common DEGs were identified by a comprehensive analysis of three expression profile databases (GSE4226, GSE4227, and GSE4229) associated with AD and NECs. GO function and KEGG pathway analyses indicated that gene expression alterations, extracellular exosome abnormalities, RNA-binding dysfunctions, and structural constituent disturbances of ribosomes were significantly enriched in AD. In addition, we used the blood samples collected from patients with AD to validate the expression of these 24 DEGs and found that 9 DEGs showed a similar change in expression as the mRNA levels of ACTG1 and ALDOA showed a significant correlation with the MoCA scores. Finally, we found that the expression of 8 of the 9 DEGs in blood and hippocampus showed the same decreased tendency in AD and the learning memory despairing aging mice (Table 2).

**Table 2:** The summary of the gene expression files from public data and validating data

		AD patients	AD mice		Aging mice	
GEO datasets		Blood	Blood	Hippocampus	Blood	Hippocampus
Upregulation	RPL23	↑	—	↓	—	↓
	RPL37A	↓	ND	ND	ND	ND
	RPL5	↓	ND	ND	ND	ND
	PPP1CB	↓	ND	ND	ND	ND
	RPL24	↓	ND	ND	ND	ND
	THRA	↓	ND	ND	ND	ND
	CASP2	↓	ND	ND	ND	ND
	SMARCD3	↓	ND	ND	ND	ND
	NLN	↓	ND	ND	ND	ND
	MSH5	↓	ND	ND	ND	ND
	EPDR1	↓	ND	ND	ND	ND
	CDH1	↓	ND	ND	ND	ND
	OTUB1	↓	ND	ND	ND	ND
	RASL11B	↓	ND	ND	ND	ND
	COMMD8	↓	ND	ND	ND	ND
	LAT2	↓	ND	ND	ND	ND
Downregulation	ACTG1	↓	↓	↓	↓	↓
	DDX5	↓	↓	↓	↓	↓
	RPS6KB2	↓	↓	↓	↓	↓
	ALDOA	↓	↓	↓	↓	↓
	NOL6	↓	↓	↓	↓	↓
	EFHD2	↓	↓	↓	↓	↓
	HERPUD1	↓	↓	↓	↓	↓
	PRRT1	↓	↓	↓	↓	↓

Upregulation: ↑, Downregulation: ↓, None detection: ND, No change: —.

Memory difficulties are one of the most common characteristics of AD and related tauopathies [21]. Analysis of both databases and AD blood sample revealed that RPL23 levels were increased in AD. RPL23 is a primary binding site between eukaryotic translation initiation factor 6 and 60S ribosomal subunit [22]. The ribosome–tau binding increases in AD compared to that in the control brains; this aberrant association leads to significantly decreased protein synthesis [23]. Ribosomal dysfunction leading to decreased translation has been implicated as an important part of AD pathogenesis. However, RPL23 expression levels in aging mice indicated a distinct regulatory function. The reason for this phenomenon is currently unclear, and thus, requires further exploration in future.

The dysfunction of the formation and plasticity of synapses is one of the pathogenic targets involved in AD [24]. EFHD2, a calcium-binding protein, is abundant in the central nervous system and directly or indirectly participates in the formation, development, and maintenance of synapses of cortical neurons [25]. Borger et al. discovered the relationship between the expression of EFHD2 and dementia through systematic and comprehensive research, and showed that both protein and mRNA levels of EFHD2 decrease in the frontal cortex of AD and other dementias, such as frontotemporal lobar degeneration. Meanwhile, the number of synapses is affected by the loss of EFHD2 [26]. The survival rate of newly formed adult neurons declines severely after EFHD2 knockout, beginning at the early stages of neuroblasts. Moreover, severe tauopathy was observed in the hippocampus of EFHD2 knockout mice [25]. This indicates that EFHD2 plays an essential role in the nervous system, and its altered expression is closely related to the occurrence and development of AD.

DEAD-box proteins in eukaryotes (37 members in humans) comprise the largest family of RNA helicases [27]; they participate in almost all aspects of RNA metabolism, including transcription, translation, and decay of RNA [28]. DDX5 (p68), a critical component of the DEAD-box family, is encoded by the *DDX5* gene located in the cell nucleus and shuttled between the nucleus and cytoplasm [29]. *DDX5* dynamically regulates the processes of transcription, splicing programs, and miRNAs during cell differentiation [30]. *DDX5* also participates in ribosome biogenesis, cell proliferation, tumorigenesis, and cancer development [31,32,33]. To date, the concrete relationship between *DDX5* and AD is unknown. This study found that *DDX5* significantly decreases in AD, and the downregulation of *DDX5* may inhibit various biological functions, such as ATP binding, hydrolysis, RNA binding and unwinding, transcription, splicing, and cell proliferation and differentiation, which may be the underlying pathological aspects of AD.

The *RPS6KB2* gene encodes a member of the ribosomal S6 kinase family of serine/threonine kinases, and phosphorylation of S6 leads to increased protein synthesis and cell proliferation [34]. Vázquez-Higuera et al. observed that compared with controls (39%), patients with AD (50%) showed a higher frequency of RPS6KB2 (intron 2, rs917570) minor allele, and the age of onset was 3 years later than that in nonminor allele carriers. Moreover, the genetic variation in the tau kinase pathway (RPS6KB2 minor allele) is related to the increased risk and later onset of AD [35]. The human *ALDOA* gene encodes the homotetrameric protein aldolase A, which is a single glycolytic enzyme in erythrocytes and skeletal muscle that catalyzes the reversible conversion of fructose-1,6-bisphosphate to glyceraldehyde 3-phosphate and dihydroxyacetone phosphate. However, to our knowledge, currently, no studies are available regarding the association between ALDOA and AD. RPS6KB2 and ALDOA were significantly downregulated in the present study in patients with AD. Moreover, ALDOA showed a significant correlation with MoCA scores. Based on the previous findings, the significant downregulation of ALDOA was assumed to lead to the reduction of aldolase A, which is an important regulator of glycolysis, which could lead to neurological abnormalities in patients with AD [36].

NOL6 (nucleolar protein 6) has been associated with ribosome biogenesis, which affects the function of many cell types in ribosome biogenesis and protein translation [37]. Previous studies regarding the relationship between NOL6 and AD involved differential expression in Mn-exposed animals in frontal cortex tissues [38], and NOL6 was identified as one of the hub genes screened from the AD database [39]. HERPUD1 (homocysteine inducible endoplasmic reticulum (ER) protein with ubiquitin like domain 1) is important in the ER stress response, which influences a number of diseases, including neurodegeneration and cardiovascular disease [40]. The expression of amyloid-β40 (Aβ40), a vital protein in AD, is decreased in HERPUD1 knockout animal models [41]. PRRT1 (proline rich transmembrane protein 1) is a candidate of α-amino-3-hydroxy-5-methyl-4-isoxazole-propionicacid receptor-associated protein as well as a component of postsynaptic density [42], which is prominently expressed in the hippocampus, particularly in CA1. PRRT1 knockout mice have weaker excitatory synapses, a loss of tetanus-induced long-term potentiation, and deficits in cognitive behaviors [43]. ACTG1 (actin gamma 1) is involved in various types of cell motility and in maintenance of the cytoskeleton, which also affects spine formation, stabilization, and morphological changes of synapses [44]. All identified DEGs in the present study showed extensive biological, neuronal, and cellular functions and may be associated with the pathophysiology of AD. Nevertheless, the functional role of these DEGs in the progress and treatment of AD requires further exploration in future.

The association of AD with aging appears to indicate that the majority of elderly people are subjected to a high probability of developing AD [45]. Common pathological factors involved in aging and AD is related to energy metabolism, inflammation, and microglial dysfunction [7]. In this study, nine DEGs reported to be associated with protein synthesis, gene transcription, and glycolysis showed the same reduced changes in AD and aging. Importantly, the decrease in the regulatory effect of these DEGs was the same in the peripheral blood and central hippocampi, indicating that they may serve as good candidates for the clinical diagnosis of AD. However, there are same bioinformatics analysis researches that reported many biomarkers for AD based on the peripheral blood mononuclear cell (PBMC) expression datasets [46,47]. Meanwhile, the currently published relevant papers were most associated with large-scale transcriptome or proteomics analysis in the brain tissues and cerebrospinal fluid samples [48,49,50]. The purpose of this study is to select potential biomarkers from GSE database and validate the expression of these DEGs in peripheral human AD samples and both peripheral/central nervous AD and aging mice samples, which could be served as both the early diagnosis biomarkers and potential targeted genes for pathological researches of AD or aging-related neurodegenerative disorders.

A comprehensive analysis of gene expression profiles was performed using systematic bioinformatics analysis to select and identify 24 common DEGs. Nine of the 24 DEGs were confirmed using blood samples collected from patients with AD, and the mRNA levels of ACTG1 and ALDOA showed a significant correlation with the MoCA scores. The levels of eight of the nine identified DEGs were decreased in both the blood and hippocampi of AD and aging mice, showing the same regulatory tendency, which provides further evidence for the reason and accuracy to detect biomarkers from blood. These eight genes could be served as both the early diagnosis biomarkers and potential targeted genes for pathological researches of AD. Our observations provided biomarkers for the early diagnosis, treatment, and prognosis of AD as well as other neurodegenerative diseases.

## Abbreviations

AD: Alzheimer’s disease
APP: amyloid precursor protein
BPs: biological processes
CCs: cellular components
DAVID: visualization and Integrated Discovery
DEGs: differentially expressed genes
ER: endoplasmic reticulum
GEO: Gene Expression Omnibus
GO: Gene Ontology
KEGG: Kyoto Encyclopedia of Genes and Genomes
MFs: molecular functions
MoCA: Montreal Cognitive Assessment
MWM: Morris water maze
NECs: normal elderly controls
PS1: presenilin 1
qPCR: quantitative real-time PCR
SA: spontaneous alternation
WT: wild-type
YM: Y maze
